# Radiographic evaluation of robot-assisted versus manual total hip arthroplasty: a multicenter randomized controlled trial

**DOI:** 10.1186/s10195-024-00773-3

**Published:** 2024-06-26

**Authors:** Xianzuo Zhang, Xianyue Shen, Rongwei Zhang, Mo Chen, Ruixiang Ma, Zian Zhang, Haining Zhang, Bo Yang, Chen Zhu

**Affiliations:** 1 Department of Orthopedics, Centre for Leading Medicine and Advanced Technologies of IHM, The First Affiliated Hospital of USTC, Division of Life Sciences and Medicine, University of Science and Technology of China, Hefei, 230001 Anhui China; 2 Department of Joint Surgery, The Affiliated Hospital of Qingdao University, Qingdao, Shandong China; 3 Department of Joint Surgery, Weifang People’s Hospital, Weifang, Shandong 261000 People’s Republic of China

**Keywords:** Robot-assisted surgery, Total hip arthroplasty, Radiographic

## Abstract

**Background:**

The effectiveness of robot-assisted surgery remains contentious due to the lack of high-quality randomized controlled trials (RCTs) to elevate the level of evidence. We aimed to evaluate the postoperative radiographic outcomes of robot-assisted (RAS-THA) versus manual (M-THA) total hip arthroplasty.

**Methods:**

This multicenter RCT was performed from March 1, 2021 to December 1, 2021. Patients were randomly assigned to routine M-THA or to RAS-THA that used the TRex-RS orthopedic joint surgical navigation system. The primary outcome was to compare the acetabular component orientation, femoral stem alignment, femoral canal fill ratio, and leg length discrepancy between RAS-THA and M-THA using postoperative radiography. Subgroup analyses of the two groups stratified by surgical approach, gender, and BMI were also conducted.

**Results:**

Seventy-three participants were randomly allocated to the RAS-THA group, while seventy-two participants were assigned to the M-THA group. Compared to the M-THA group, the RAS-THA group exhibited less variability in the preoperative planning of the vertical center of rotation (VCOR; *P* < 0.001), demonstrated a significant advantage in femoral stem alignment (*P* = 0.004), and showed pronounced decreases in inequality and in the variability in leg length discrepancy (*P* < 0.001). There was no significant difference in the Lewinnek safe-zone ratio (*P* = 0.081) and the femoral canal fill ratio (*P* > 0.05) between the two groups. Further subgroup analysis also showed that the RAS-THA group had fewer horizontal center of rotation (HCOR) and leg length differences when stratified by surgical approach, gender, and overweight status.

**Conclusion:**

This RCT found that, regardless of the surgical approach, gender, or body mass index, RAS-THA can effectively improve the postoperative VCOR and significantly reduce the variability of leg length difference. RAS-THA should be considered an effective method to enhance surgical precision by achieving less variability in challenging patients with leg length discrepancies.

*Trial registration*: ChiCTR2100044124.

**Supplementary Information:**

The online version contains supplementary material available at 10.1186/s10195-024-00773-3.

## Introduction

The escalating incidence of hip degenerative diseases has led to a surge in the demand for total hip arthroplasty (THA). In the United States alone, an estimated 3.7 million THAs are performed annually, with projections indicating a rise to between 572,000 and 635,000 primary THAs by 2030 [1, 2]. While THA has garnered substantial acclaim for its clinical efficacy [3], the success of the procedure is contingent upon the meticulous placement of hip components. Emerging technologies, such as robot-assisted THA (RAS-THA), have been introduced to augment the precision of these key surgical steps.

Initially conceived in the 1990s, RAS-THA has undergone significant advancements to enhance the accuracy and reproducibility of orthopedic surgeries [4, 5]. Over the past decade, the adoption of RAS-THA has seen a linear increase, offering potential advantages in relation to component positioning, limb length discrepancies, and reduced complications [6–8]. However, the comparative effectiveness of RAS-THA and manual THA (M-THA) remains a subject of ongoing debate [9]. While some studies advocate for the superiority of RAS-THA in specific metrics [6–8], conflicting evidence exists regarding its overall clinical benefits and cost-effectiveness [10, 11].

Given the prevailing reliance on small-scale retrospective cohort studies [8, 12–15], there is an exigent need for a robust, multicenter randomized controlled trial (RCT) to substantiate the evidence base for RAS-THA. To this end, we initiated an open-label, parallel-group, multicenter RCT across three institutions in China. The primary aim of this study is to evaluate the postoperative radiographic outcomes of RAS-THA in comparison to M-THA for treating diverse hip pathologies. Secondary objectives include the assessment of clinically significant differences stratified by surgical approach, gender, and body mass index (BMI) between the two groups.

## Materials and methods

### Study design and participants

This study was a prospective, multicenter, open-label, parallel, non-inferiority RCT conducted from March 1, 2021 to December 1, 2021. Participants were recruited from three institutions in China: the First Affiliated Hospital of USTC, the Affiliated Hospital of Qingdao University, and Weifang People's Hospital. Ethical approval was obtained from the institutional review boards of the participating hospitals, and all participants provided written informed consent. The trial was conducted in accordance with the International Conference on Harmonization Guidelines for Good Clinical Practice and the Declaration of Helsinki. The trial was registered with clinicaltrials.gov (ChiCTR2100044124) and adhered to the Consolidated Standards of Reporting Trials (CONSORT) guidelines. Eligible subjects were individuals aged 18–75 years who presented with end-stage hip disease requiring THA and were willing to adhere to trial protocols. Exclusion criteria are detailed in eTable 1 and eTable 2 in the supplementary information.

### Randomization and masking

Participants were informed about the trial’s standardized procedures either orally or in writing. The stratified blocked randomization method was adopted in the trial. A researcher who was blinded to the study groups used statistical software to generate a random list of assignments. This was done by formulating random seeds, determining block sizes, and stratifying the assignments by center. The patients were randomly assigned in a 1:1 ratio to receive either THA assisted with the TRex-RS (version HIP 1.0) orthopedic joint surgical navigation system (Longwell Company, Shanghai, China) (intervention group; RAS-THA) or freehand THA performed by orthopedic surgeons (control group; M-THA). The clinicians responsible for conducting the subsequent evaluations were blinded to the group allocations.

### Intervention

Patients assigned to the control group underwent routine THA, with the surgical technique following the technical manual provided by the manufacturer of the hip joint prosthesis. The intervention group underwent RAS-THA using TRex-RS, a semi-active surgical robot that generates a three-dimensional (3D) model from preoperative computed tomography (CT) to assist the surgeon in selecting the most appropriate prosthetic model and size and improve implant placement. All procedures used a cementless prosthesis, and surgery was performed under general anesthesia unless contraindicated. The target inclination and anteversion of the acetabular component were 40° and 15°, respectively. The components were positioned with the same target in the two groups. The procedures were performed by senior hip surgeons at each center, each of whom performed more than 100 THAs annually. The choice of operation approach depended on the surgeon’s preference and mainly included a direct anterior approach, lateral approach, and posterior approach. The workflow of RAS-THA is reported in the eMethods in the supplementary information.

### Postoperative rehabilitation management

All patients followed the same standardized rehabilitation program at a particular time postoperatively. Both groups were treated with pain relief, thrombosis prevention, swelling reduction, and prophylactic antibiotics after the operation. All clinical data were stored in electronic medical records throughout the study period.

### Outcome measures

The primary outcomes included four domains: acetabular component orientation, femoral stem alignment, femoral canal fill ratio, and leg length discrepancy. Details of radiographic parameter measurements are presented in the eMethods in the supplementary information.

### Sample size

According to literature reports and clinical experience [8], the implantation success rate in the control group is 96.1%. We assume that the implantation success rate of the test group is not inferior to that of the control group, and the non-inferiority margin was set as −10%. The number of test subjects was at least 118 cases (59 cases in the test group and 59 cases in the control group). Based on a one-sided type I error of 2.5% and a dropout rate of 20% and to meet the needs of the block random design, the number of subjects should be at least 148 to achieve a test efficiency power of 80%. Considering the influence of center differences, in order to ensure the representativeness of the subjects in each clinical trial institution, in principle, the number of subjects in each clinical trial institution should be 20–60% of the overall sample size.

### Statistical analysis

Categorical variables were reported as the frequency and percentage, while continuous data were reported as the mean and standard deviation (SD). Group differences between RAS-THA and M-THA were compared using independent* t* tests for continuous variables, and categorical variables were analyzed using *χ*^2^ or Fisher's exact tests as appropriate. Prespecified subgroup analyses included those focusing on the surgical approach, the patient's body mass index (obesity), and the patient’s sex. All the statistical analyses were performed at the two-sided 5% significance level and conducted using SPSS software version 23 (SPSS Inc., Chicago, IL, USA).

## Results

### Participant characteristics

**Fig. 1:**
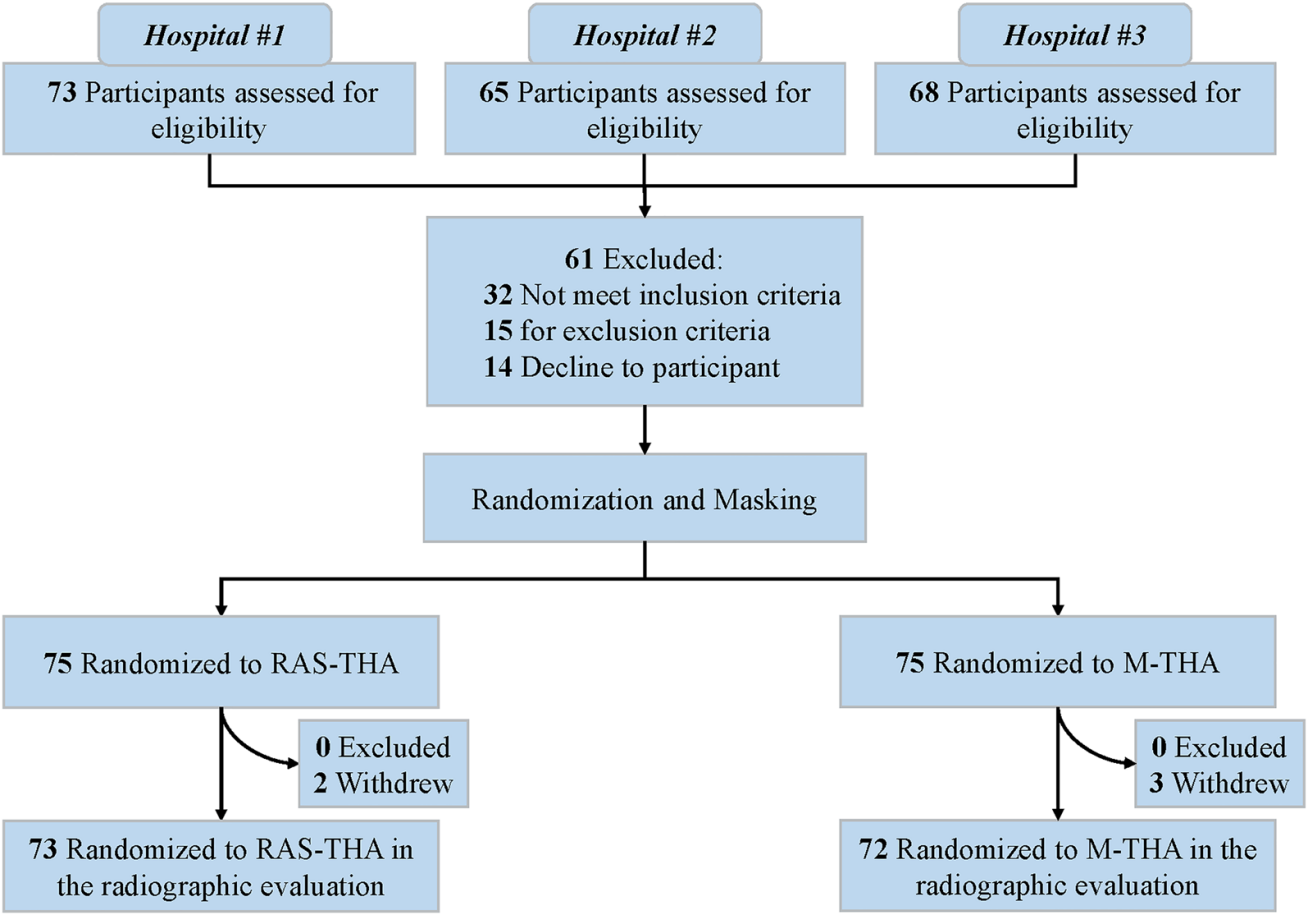
CONSORT flowchart of participants through the randomized controlled trial

Participant recruitment took place between March 2021 and December 2021 at the orthopedic centers of three hospitals. A total of 145 participants with a mean age of 56.6 ± 9.9 years who met the inclusion criteria were enrolled (Fig. 1). Of these, 79 were male and 66 were female. Among the 145 eligible patients, 73 were randomly allocated to the RAS-THA group, while 72 participants were assigned to the M-THA group through randomization. Baseline demographic and clinical characteristics were well balanced between the treatment groups (Table 1). The primary hip-related pathology among the included patients was femoral head necrosis, followed by developmental dysplasia of the hip (DDH).

**Table 1:** Demographics and baseline characteristics

Characteristics	Overall (*n* = 145)	RAS-THA (*n* = 73)	M-THA (*n* = 72)	*P* value
Sex				0.208
Male	79 (54.5%)	36 (49.3%)	43 (59.7%)	
Female	66 (45.5%)	37 (50.7%)	29 (40.3%)	
Age in years, mean (SD)	56.6 ± 9.9	58.1 ± 9.0	55.0 ± 10.7	0.063
< 40	11 (7.6%)	3 (4.1%)	8 (11.1%)	
40–49	20 (13.8%)	10 (13.7%)	10 (13.9%)	
50–59	57 (39.3%)	27 (37.0%)	30 (41.7%)	
60–69	42 (30.0%)	24 (32.9%)	18 (25.0%)	
70–79	15 (10.3%)	9 (12.3%)	6 (8.3%)	
Surgical side				0.113
Left	77 (53.1%)	34 (46.6%)	43 (59.7%)	
Right	68 (46.9%)	39 (53.4%)	29 (40.3%)	
Body mass index	25.1 ± 3.6	25.1 ± 3.55	25.2 ± 3.6	0.928
Diagnosis				
ONFH	103 (71.0%)	48 (65.8%)	55 (76.4%)	
DDH	24 (16.6%)	15 (20.5%)	9 (12.5%)	
OA	12 (8.3%)	6 (8.2%)	6 (8.3%)	
FNF	3 (2.1%)	3 (4.1%)	0 (0.0%)	
AS	3 (2.1%)	1 (1.4%)	2 (2.8%)	

*RAS-THA* robot-assisted total hip arthroplasty, *M-THA* manual total hip arthroplasty, *SD* standard deviation, *OA* osteoarthritis, *FNF* femoral neck fracture, *ONFH* osteonecrosis of the femoral head, *DDH* developmental dysplasia of the hip, *AS* ankylosing spondylitis

## Primary outcomes

### Acetabular component orientation

**Fig. 2:**
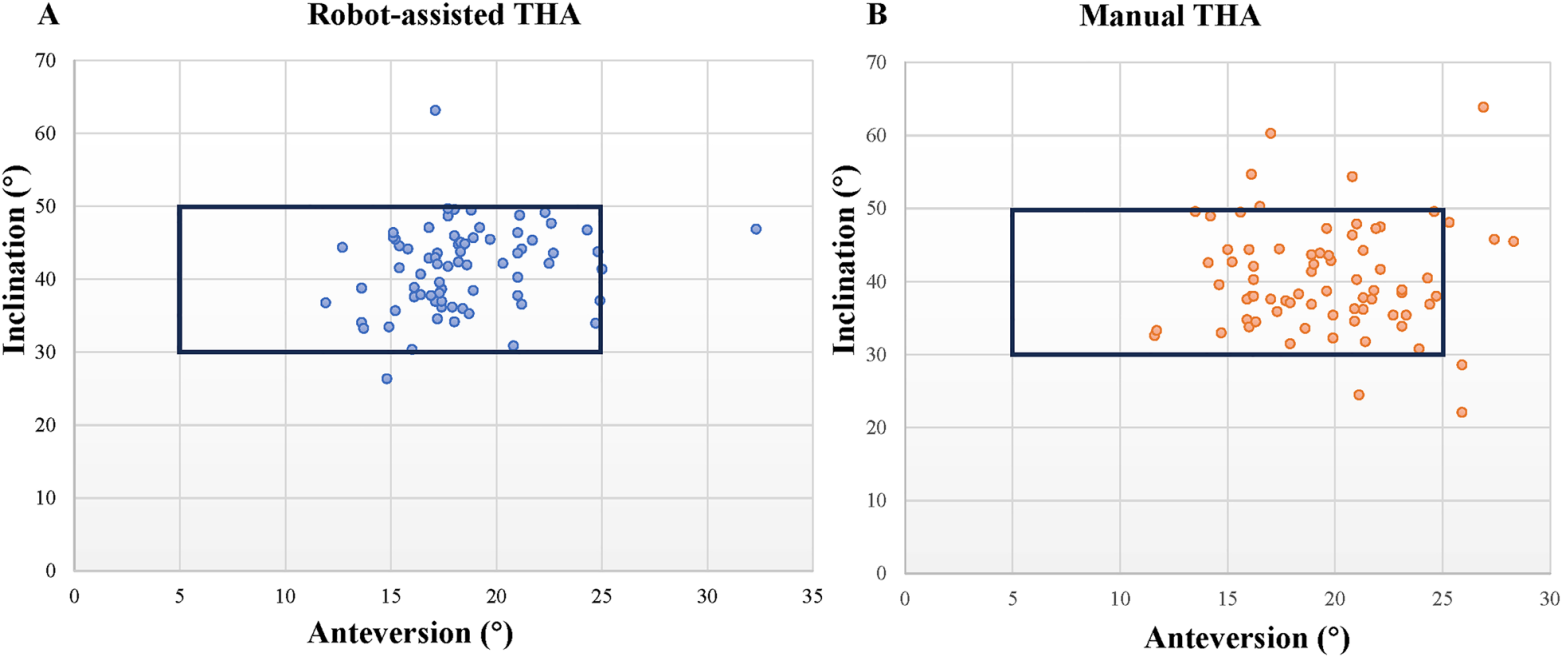
Distribution of acetabular component positions for the robot-assisted THA group (**a**) and the manual THA group (**b**). The region within the black box area (between 30° and 50° for inclination and between 5° and 25° for anteversion) represents the Lewinnek safe zone

In the RAS-THA group, the horizontal center of rotation (HCOR) and vertical center of rotation (VCOR) from the preoperative predetermined COR were − 0.04 ± 1.30 and 0.24 ± 1.36 mm, respectively. In the M-THA group, the corresponding values were − 0.14 ± 6.13 and 7.10 ± 13.06 (Table 2). The two groups only showed significant differences in VCOR (*P* < 0.001). The inclination and anteversion of the implanted cup were not significantly different between the RAS-THA and M-THA groups (*P* = 0.298, *P* = 0.071). In addition, among the 73 RAS-THA patients, 72 (97.3%) had the cup located in the Lewinnek safe zone, while 65 patients in the M-THA group had the cup located in the Lewinnek safe zone, with no statistical difference between the two groups (*P* = 0.081) (Fig. 2).

**Table 2:** Comparison of the postoperative radiographic results between robot-assisted THA and manual THA

Parameter	RAS-THA	M-THA	Effect size (95% CI)	*P* value
Domain 1: acetabular component orientation				
HCOR (mm)	−0.04 ± 1.3	−0.14 ± 6.1	−1.36 to 1.58	0.885
VCOR (mm)	0.24 ± 1.4	7.1 ± 13.1	−9.94 to −3.77	< 0.001**
Inclination (°)	41.6 ± 5.8	40.4 ± 7.4	−1.03 to 3.34	0.298
Anteversion (°)	18.5 ± 3.4	19.5 ± 3.8	−2.27 to 0.09	0.071
Lewinnek’s safe zone (%)	71/73	65/72	–	0.081
Domain 2: femoral stem alignment				
Femoral stem alignment (°)	1.8 ± 0.6	2.2 ± 1.1	−0.75 to −0.15	0.004**
Domain 3: femoral canal fill ratio				
Coronal osteotomy site (%)	61.1 ± 10.9	62.8 ± 8.5	−4.97 to 1.49	0.29
Sagittal osteotomy site (%)	93.5 ± 11.6	95.3 ± 3.8	−4.70 to 0.99	0.20
Coronal osteotomy site at 2.5 cm (%)	77.5 ± 12.9	78.1 ± 9.0	−4.30 to 3.04	0.73
Sagittal osteotomy site at 2.5 cm (%)	93.2 ± 11.6	94.2 ± 4.0	−3.93 to 1.80	0.46
Coronal osteotomy site at 7.5 cm (%)	75.7 ± 19.7	74.3 ± 19.7	−5.06 to 7.87	0.67
Sagittal osteotomy site at 7.5 cm (%)	55.6 ± 18.2	56.1 ± 16.8	−6.26 to 5.25	0.86
Coronal osteotomy site at isthmus (%)	70.1 ± 20.9	67.7 ± 22.2	−4.72 to 9.43	0.51
Sagittal osteotomy site at isthmus (%)	50.9 ± 18.0	49.9 ± 18.3	−4.91 to 7.00	1.04
Domain 4: leg length discrepancy				
Leg length discrepancy (mm)	2.9 ± 1.5	5.8 ± 6.3	−4.48 to −1.44	< 0.001**
Equal leg	72/73	50/72	–	< 0.001**

*RAS-THA* robot-assisted total hip arthroplasty, *M-THA* manual total hip arthroplasty, *CI* confidence interval, *HCOR* horizontal displacement of the acetabular center of rotation, *VCOR* vertical displacement of the acetabular center of rotation

* represents *P* < 0.05, ** represents *P* < 0.01

### Femoral stem alignment

By measuring the angle between the long axis of the femoral stem and the femoral axis on immediate postoperative anteroposterior X-rays, it was found that the angle between the femoral and prosthetic axes was significantly larger in the M-THA group compared to the RAS-THA group (1.8° ± 0.6° vs. 2.2° ± 1.1°, *P* = 0.004).

### Femoral canal fill ratio

The femoral canal fill ratio in the coronal and sagittal planes was calculated at the selected four femoral osteotomy sites to evaluate the effectiveness of proximal and distal fixation of the femoral stem prosthesis. The results showed that there was no statistically significant difference in the femoral canal fill ratio between the RAS-THA and M-THA groups at each site, whether in the coronal or the sagittal plane (Table 2).

### Leg length discrepancy

Leg length discrepancies were assessed through radiographic measurements. The results revealed a leg length discrepancy of 2.9 ± 1.5 mm in the RAS-THA group, whereas it was 5.8 ± 6.3 mm in the M-THA group. The RAS-THA group demonstrated a significant advantage due to its smaller postoperative leg length discrepancy compared to M-THA (*P* < 0.001). When the difference is not greater than 5 mm, the legs are considered to be equal in length, and the proportion of equal leg lengths after RAS-THA is significantly higher than that of M-THA (98.6% vs. 69.4%, *P* < 0.001).

### Sensitivity analysis

The present study underwent a subgroup exploration in terms of gender, overweight status (BMI ≥ 24), and surgical approach. When subjects were stratified by gender, among male participants, VCOR (*P* < 0.001), anteversion (*P* = 0.047), femoral stem alignment (*P* = 0.049), and leg length discrepancy (*P* = 0.010) were all significantly better in the RAS-THA group than in the M-THA group. Among female participants, however, the RAS-THA group showed significant differences from the M-THA group in terms of HCOR (*P* = 0.031) and VCOR (*P* = 0.034), inclination (*P* = 0.009), femoral stem alignment (*P* = 0.039), and leg length discrepancy (*P* = 0.011) (eTable 3 in the supplementary information). The results of the subgroup analysis also showed that in the overweight population, the RAS-THA group revealed a smaller VCOR (*P* = 0.007) and superior leg length discrepancy (*P* = 0.027) compared to the M-THA group. In the non-overweight population, RAS-THA showed a smaller VCOR (*P* = 0.002), femoral stem alignment (*P* = 0.026), and leg length discrepancy (*P* = 0.004) (eTable 4 in the supplementary information). In the study, we mainly compared the radiographic performances of the direct anterior approach and the posterior or lateral approaches in RAS-THA and M-THA, respectively. In the context of the anterior approach, RAS-THA only showed advantages in VCOR (*P* = 0.002), anteversion (*P* = 0.026), femoral stem alignment (*P* = 0.010), and leg length discrepancy (*P* = 0.006); in the posterior and lateral approaches, the differences between the two groups were mainly reflected in the VCOR (*P* = 0.034), inclination (*P* = 0.009), and leg length discrepancy (*P* = 0.014) (eTable 3 in the supplementary information).

## Discussion

This study contributes to the existing literature by being a prospective, multicenter RCT that rigorously evaluates the postoperative radiographic outcomes of RAS-THA and M-THA for hip-related pathologies. Our findings suggest a notable advantage for RAS-THA in terms of VCOR, femoral stem alignment, and leg length discrepancy when compared to M-THA. These advantages were consistent across various subgroups, including different genders, BMI categories, and surgical approaches, thereby supporting the potential of RAS-THA as a reliable and precise option for THA, especially in the restoration of anatomical hip-joint function.

Despite THA being considered one of the most successful surgeries of the twenty-first century [3, 16], clinical complications related to component positioning persist, such as deficits in acetabular cup orientation and COR reconstruction that lead to dislocation and muscle strength problems [17–19]. Robot-assisted technology has been extensively adopted in orthopedic surgery, as it demonstrates significant potential advantages [20–23]. It claims to improve accuracy and precision in the preparation of bone surfaces, enabling precise acetabular reaming and accurate component placement, which has made it a technique that is perceived to be reliable and reproducible. Nevertheless, the efficacy of RAS-THA in improving prosthesis positioning remains a subject of debate [8, 24]. In the trial, the results revealed no significant differences in the anteversion and inclination of the acetabular component between the two groups. These two metrics are crucial determinants when establishing the optimal positioning of the acetabular component, as proper placement can effectively reduce the risks of hip instability, impingement, and restricted joint mobility. Furthermore, our results demonstrated no significant difference in the cup placement rate within the Lewinnek safe zone between the two groups, which was consistent with the findings of Guo et al. [25]

However, there are also some divergent viewpoints that differ from those of Kong et al. [26], who found that the cup inclination and safety zone ratio of RAS-THA were superior to those of M-THA in a retrospective study. We speculate that the associated differences may be attributable to differences in study design and surgical robotic systems. Further, the trial analyzed the effectiveness of two surgical techniques in reconstructing the hip center of rotation, which generally ensures good muscle tone and longevity of the prosthesis. Our results showed that traditional M-THA would cause the COR to move up by about 7.1 mm, while this figure was 0.2 mm in RAS-THA, showing a high coincidence with the preset COR. This may suggest that robot-assisted reaming of the acetabular technique may be beneficial. Our findings were also confirmed in subgroup explorations.

Leg length discrepancy remains a significant postoperative concern, as it often leads to gait abnormalities and reduced patient satisfaction [27]. Our study demonstrated a significant advantage for RAS-THA in minimizing leg length discrepancy, which is supported by prior research. For instance, in a comparative study, Guo et al. [25] found that the leg length discrepancy of the RAS-THA group was significantly smaller than that of the M-THA group. A prospective study by Honl et al. [28] found that the leg length discrepancy was significantly smaller in RAS-THA using the five-axis ROBODOC system compared with M-THA. In contrast, Kayani et al. [29] compared 25 patients undergoing posterolateral RAS-THA with 50 patients undergoing conventional M-THA by the same surgeon and found no difference in achieving leg length correction. This difference is mainly attributed to the small sample size of the study, which may have biased the conclusions. However, in this trial, the sample of research subjects was larger, the research design was more rigorous, and the relevant results were also verified in further subgroup analyses. This result is exciting, especially for those patients with huge differences in the lengths of the lower limbs due to hip diseases, such as collapsed osteonecrosis of the femoral head and developmental dysplasia of the hip, as it can correct the leg length and restore the patient's lower limb length more accurately.

Our study did not find significant differences in femoral canal fill ratios between the groups, indicating that both manual and robotic techniques are effective. However, the precision of femoral stem alignment was notably better in the RAS-THA group, suggesting that robot-assisted technology, especially when utilizing preoperative 3D CT, could offer personalized femoral stem placement and reduce varus or valgus alignment issues. An abnormal varus–valgus angle of the femoral stem may affect the hip offset, causing additional pressure or friction on the surrounding tissue, which causes persistent discomfort or thigh pain for the patient and ultimately shortens the life of the component [30–32]. In terms of femoral canal fill, these results indicate that both the manual selection and the robotic selection of femoral stem size are accurate, but in the process of femoral reaming, robot-assisted technology using preoperative 3D CT can effectively provide a personalized template and then perform virtual projection during the surgery according to the mapped bone landmarks, which can accurately provide personalized femoral stem placement and reduce stem varus or valgus.

## Strengths and limitations

The strengths of this trial lie in its open-label, multicenter randomized controlled design and the participating institutions (three hospitals in China), which ensure the representativeness and generalization of the results. In addition, all surgeons received uniform training in standardized arthroplasty procedures to minimize heterogeneity among surgical techniques, and the same inclusion and exclusion criteria were applied to all subjects. With this rigorous methodological design, we believe that the results of this trial add new evidence supporting the use of RAS-THA in promoting postoperative femoral stem alignment and reducing leg length discrepancy, thereby improving clinically important outcomes that previously lacked high-quality evidence.

Several limitations of the trial are worth noting. Firstly, this study mainly focused on a postoperative radiographic evaluation and was unable to compare postoperative clinical functional outcomes and complications between the two groups of participants. This is mainly due to the fact that the follow-up is still in progress, but for THA, the radiographic performance is usually one of the key indicators of surgical efficacy. Second, a multicenter design involves several participating surgeons, and the technique may be less standardized than a proof-of-concept design within a single center. Nevertheless, the rich surgical experience and the surgical capacity ensured that the surgeons performed safe THA and the implementation of uniform techniques. Of course, the trial should be considered realistic and reflective of common clinical practice, so the results should be generalizable to a wider patient population with characteristics similar to those included in the trial. Third, this trial did not take into account the operative time and additional costs, which affected the appropriate cost range, and robot-assisted technology must be within the appropriate economics to justify its widespread use.

## Conclusion

In conclusion, this randomized clinical trial demonstrated that RAS-THA effectively improved the postoperative VCOR and significantly reduced the variability in leg length discrepancy, regardless of the surgical approach, gender, or patient BMI. This information confirms and expands the evidence that robot-assisted technology can improve surgical precision. RAS-THA should be considered as an effective adjunct to achieve low variability in leg length differences in challenging patients.

### Supplementary information

Supplementary material 1.

## Data Availability

All the data in the study are available from the corresponding author on reasonable request.
