# Supplementary material for: Radiographic evaluation of robot-assisted versus manual total hip arthroplasty: a multicenter randomized controlled trial
Source: J Orthop Traumatol. 2024 Jun 26;25:33. doi: 10.1186/s10195-024-00773-3 (PMC11208392; doi:10.1186/s10195-024-00773-3)
Supplement: Supplementary file 1 — Supplementary material 1. [file 10195_2024_773_MOESM1_ESM.docx]

Supplementary files:

eMothods

eTable1. Detailed exclusion criteria for the randomized controlled trial.

eTable2. Detailed withdrawal criteria for the randomized controlled trial.

eTable3. Postoperative radiographic of robot-assisted THA versus manual THA when stratified by gender.

eTable4. Postoperative radiographic of robot-assisted THA versus manual THA when stratified by body mass index.

eTable5. Postoperative radiographic of robot-assisted THA versus manual THA when stratified by surgical approach.

eFigure1. Schematic diagram of measurement of radiographic parameters.

**eMethods**

**Intervention**

The workflow of robotic-assisted total hip arthroplasty (RAS-THA): After robotic arm registration, two positioning pins are first inserted into the iliac crest, which are used to fix the universal tracker. Then, the spatial position of the femur calibrated by the optical tracker and the morphology of the femur in the computed tomography (CT) scan were registered through the steps of coarse registration and fine registration. Once femur registration is complete, the osteotomy line is determined with a probe and the femoral head is removed with a saw under the guidance of the robotic arm. Subsequently, the acetabulum is registered and the acetabulum is reamed with the assistance of the robotic arm, followed by implantation of the recommended acetabular component size based on software recommendation, with additional screws optionally used for initial fixation. After completing the acetabulum operation, the workpiece can be changed to perform the femoral medullary cavity opening and proximal and distal femoral milling, followed by the implantation of the femoral stem with the assistance of the robotic arm. Once the implant is in place, the anatomical position parameters of the acetabular and femoral components are calculated, and the stability and range of motion of the hip joint are tested. All information can be fed back in real-time on the screen for verifying the surgical outcome.

**Outcome measures**

In this trial, four radiographic indicators were mainly selected to evaluate the acetabular component orientation, including inclination, anteversion, horizontal center of rotation displacement (HCOR) and vertical displacement COR (VCOR). In the standard hip anteroposterior X-ray, acetabular component inclination and anteversion was calculated using Pradhan’s method[1]. The horizontal and vertical differences between the postoperative COR and preoperative designed COR are designed as HCOR and VCOR (eFigure 1). A line is made along the lowest point of the bilateral ischial tuberosity on the standard hip anteroposterior X-ray, and the vertical distance from the bilateral femoral trochanter to this line is considered as leg length discrepancy (eFigure 1). The femoral stem alignment is mainly evaluated by the valgus and varus angle of the femoral prosthesis, which is determined by measuring the angle between the long axis of the stem and the long axis of the femur in the standard hip anteroposterior X-ray (eFigure 1). In addition, according to Kobayashi’s study[2], the ratio of the stem diameter to the femur canal diameter was calculated as the stem-canal ratio.

All patients underwent bilateral hip anteroposterior and weight-bearing full-length lower limb radiographic examinations before surgery. In addition, further radiographic examination within 3±1 day after surgery was also performed. All images were extracted and desensitized from the Picture Archiving and Communication Systems workstation and exported in Digital Imaging and Communications in Medicine (DICOM) format. Radiographic measurements were performed using an independent blinded radiographic review panel to minimize bias in radiographic variables. Categorical variables were assessed independently by two experienced and qualified orthopedic surgeons according to standardized criteria. In the event of any discrepancies between the assessments, the final decision was made by a senior orthopedic surgeon. Continuous variables were measured by two experienced and qualified orthopedic surgeons, and the mean value was taken as the final result.

**Reference:**

[1] Lee GC, Lee SH, Kang SW, Park HS, Jo S. Accuracy of planar anteversion measurements using anteroposterior radiographs. BMC Musculoskelet Disord. 2019 Dec 5;20(1):586. doi: 10.1186/s12891-019-2979-0.

[2] Kobayashi S, Eftekhar NS, Terayama K. Predisposing factors in fixation failure of femoral prostheses following primary Charnley low friction arthroplasty. A 10- to 20-year followup study. Clin Orthop Relat Res. 1994 Sep;(306):73-83.

| Detailed exclusion criteria: |
| --- |
| 1. Patients who are allergic to the drug or implant materials in this trial |
| 1. Skeletally immature patients |
| 1. Patients with active infection |
| 1. Patients with hip neuropathy |
| 1. Poor bone quality is not conducive to the fixation of biological prosthesis |
| 1. General conditions or concomitant diseases make it difficult for people to tolerate surgery |
| 1. Patients with hip weakness |
| 1. Patients with morbid obesity, body mass index (BMI) >35 |
| 1. Insufficient bone mass of the greater trochanter or insufficient skin coverage nearby, and patients who cannot tolerate surgery |
| 1. Patients with severe femoral or pelvic deformities and other severe extra-articular deformities |
| 1. Metal implants in the patient's hip joint surgical area affect the execution of the surgical plan |
| 1. Pregnant or lactating patients |
| 1. Patients with severe epilepsy or mental illness |
| 1. Alcohol or drug dependent patients |
| 1. Patients with severe coagulation disorders |
| 1. Patients who have participated in or are currently participating in other clinical trials within one month of enrollment |
| 1. Patients who were rejected by the investigator for other reasons in this clinical trial |

eTable1. Detailed exclusion criteria for the randomized controlled trial.

eTable2. Detailed withdrawal criteria for the randomized controlled trial.

| Detailed withdrawal criteria: |
| --- |
| 1. Subjects withdraw informed consent |
| 1. Subjects seriously violated the clinical trial protocol |
| 1. Investigators believe that it is no longer suitable to continue clinical trials |
| 1. Subjects who become pregnant during clinical trials |
| 1. Subjects died during clinical trial |
| 1. Subject loss of follow-up |

eTable3. Postoperative radiographic of robot-assisted THA versus manual THA when stratified by gender.

| Parameter | RAS-THA | M-THA | *P* value |
| --- | --- | --- | --- |
| **Male** | | | |
| HCOR (mm) | -0.12±1.38 | 1.32±6.15 | 0.143 |
| VCOR (mm) | 0.46±1.82 | 8.5±13.69 | ＜0.001** |
| Inclination (°) | 39.65±5.18 | 41.12±8.03 | 0.348 |
| Anteversion (°) | 19.60±4.18 | 20.03±3.46 | 0.047* |
| Coronal osteotomy site (%) | 60.09±8.42 | 61.60±9.42 | 0.475 |
| Sagittal osteotomy site (%) | 94.89±3.25 | 95.60±4.13 | 0.410 |
| Coronal osteotomy site at 2.5cm (%) | 77.29±9.79 | 76.87±10.22 | 0.853 |
| Sagittal osteotomy site at 2.5cm (%) | 95.00±3.30 | 94.23±4.34 | 0.383 |
| Coronal osteotomy site at 7.5cm (%) | 80.29±7.33 | 79.63±7.07 | 0.693 |
| Sagittal osteotomy site at 7.5cm (%) | 58.20±13.13 | 58.32±10.50 | 0.965 |
| Coronal osteotomy site at isthmus (%) | 73.18±14.93 | 73.69±8.80 | 0.855 |
| Sagittal osteotomy site at isthmus (%) | 52.51±15.35 | 53.61±11.12 | 0.721 |
| Femoral stem alignment (°) | 1.83±0.65 | 2.28±1.22 | 0.049* |
| Leg length discrepancy (mm) | 3.10±1.77 | 5.87±6.52 | 0.010** |
| **Female** | | | |
| HCOR (mm) | 0.04±1.28 | -2.31±5.51 | 0.031* |
| VCOR (mm) | 0.02±0.60 | 5.00±12.00 | 0.034* |
| Inclination (°) | 43.40±5.82 | 39.32±6.43 | 0.009** |
| Anteversion (°) | 19.21±3.68 | 19.98±3.52 | 0.394 |
| Coronal osteotomy site (%) | 63.80±6.77 | 64.66±6.80 | 0.611 |
| Sagittal osteotomy site (%) | 94.63±3.80 | 94.91±3.25 | 0.760 |
| Coronal osteotomy site at 2.5cm (%) | 79.86±8.43 | 80.00±6.63 | 0.942 |
| Sagittal osteotomy site at 2.5cm (%) | 93.90±4.03 | 94.21±3.39 | 0.737 |
| Coronal osteotomy site at 7.5cm (%) | 79.93±7.41 | 77.24±8.81 | 0.206 |
| Sagittal osteotomy site at 7.5cm (%) | 59.33±12.23 | 60.98±9.31 | 0.561 |
| Coronal osteotomy site at isthmus (%) | 75.02±9.89 | 74.18±8.92 | 0.737 |
| Sagittal osteotomy site at isthmus (%) | 55.16±11.42 | 55.78±10.19 | 0.825 |
| Femoral stem alignment (°) | 1.74±0.63 | 2.15±0.94 | 0.039* |
| Leg length discrepancy (mm) | 2.66±1.27 | 5.80±6.08 | 0.011** |

RAS-THA, robot-assisted total hip arthroplasty; M-THA, manual total hip arthroplasty; HCOR, horizontal displacement of the acetabular center of rotation; VCOR, vertical displacement of the acetabular center of rotation. * represents P<0.05, ** represents P<0.01.

eTable4. Postoperative radiopraphic of robot-assisted THA versus manual THA when stratified by body mass index.

| Parameter | RAS-THA | M-THA | *P* value |
| --- | --- | --- | --- |
| **BMI≥24** | | | |
| HCOR (mm) | -0.03±1.2 | -1.02±4.62 | 0.18 |
| VCOR (mm) | 0.15±0.92 | 5.21±11.92 | 0.007** |
| Inclination (°) | 41.47±6.48 | 39.90±6.79 | 0.274 |
| Anteversion (°) | 17.74±2.62 | 19.16±3.94 | 0.051 |
| Coronal osteotomy site (%) | 62.20±8.42 | 63.32±8.64 | 0.547 |
| Sagittal osteotomy site (%) | 94.63±3.90 | 95.30±4.15 | 0.442 |
| Coronal osteotomy site at 2.5cm (%) | 79.11±9.04 | 78.68±9.19 | 0.826 |
| Sagittal osteotomy site at 2.5cm (%) | 94.91±3.44 | 94.60±4.02 | 0.698 |
| Coronal osteotomy site at 7.5cm (%) | 79.67±7.46 | 78.30±8.00 | 0.419 |
| Sagittal osteotomy site at 7.5cm (%) | 59.20±13.09 | 59.15±10.32 | 0.985 |
| Coronal osteotomy site at isthmus (%) | 74.90±9.59 | 73.69±8.44 | 0.545 |
| Sagittal osteotomy site at isthmus (%) | 55.40±12.52 | 55.10±11.06 | 0.906 |
| Femoral stem alignment (°) | 1.70±0.65 | 1.95±0.87 | 0.123 |
| Leg length discrepancy (mm) | 3.14±1.74 | 5.39±6.33 | 0.027** |
| **BMI＜24** | | | |
| HCOR (mm) | -0.08±1.51 | 1.19±7.57 | 0.375 |
| VCOR (mm) | 0.39±1.88 | 9.37±14.14 | 0.002** |
| Inclination (°) | 41.81±4.64 | 41.07±8.26 | 0.673 |
| Anteversion (°) | 19.60±4.18 | 20.03±3.46 | 0.668 |
| Coronal osteotomy site (%) | 62.20±8.42 | 63.32±8.64 | 0.771 |
| Sagittal osteotomy site (%) | 94.63±3.90 | 95.30±4.15 | 0.752 |
| Coronal osteotomy site at 2.5cm (%) | 62.16±7.55 | 61.53±8.97 | 0.443 |
| Sagittal osteotomy site at 2.5cm (%) | 95.03±2.94 | 95.29±3.23 | 0.857 |
| Coronal osteotomy site at 7.5cm (%) | 78.52±8.75 | 76.67±9.47 | 0.172 |
| Sagittal osteotomy site at 7.5cm (%) | 57.78±12.05 | 60.06±9.75 | 0.453 |
| Coronal osteotomy site at isthmus (%) | 73.14±16.45 | 73.95±9.37 | 0.829 |
| Sagittal osteotomy site at isthmus (%) | 50.66±14.15 | 54.16±10.96 | 0.324 |
| Femoral stem alignment (°) | 1.95±0.61 | 2.58±1.32 | 0.026* |
| Leg length discrepancy (mm) | 2.58±1.05 | 6.20±6.25 | 0.004** |

RAS-THA, robot-assisted total hip arthroplasty; M-THA, manual total hip arthroplasty; BMI, body mass index; HCOR, horizontal displacement of the acetabular center of rotation; VCOR, vertical displacement of the acetabular center of rotation. * represents P<0.05, ** represents P<0.01.

eTable5. Postoperative radiographic of robot-assisted THA versus manual THA when stratified by surgical approach.

| Parameter | RAS-THA | M-THA | *P* value |
| --- | --- | --- | --- |
| **Direct anterior approach** | | | |
| HCOR (mm) | -0.28±1.30 | -0.96±4.86 | 0.354 |
| VCOR (mm) | 0.38±1.61 | 6.58±13.15 | 0.002** |
| Inclination (°) | 41.23±6.26 | 39.80±7.77 | 0.319 |
| Anteversion (°) | 18.52±3.71 | 20.26±3.86 | 0.026* |
| Coronal osteotomy site (%) | 62.17±8.53 | 64.34±8.93 | 0.226 |
| Sagittal osteotomy site (%) | 94.85±3.44 | 95.89±3.96 | 0.177 |
| Coronal osteotomy site at 2.5cm (%) | 80.54±8.00 | 80.07±8.79 | 0.783 |
| Sagittal osteotomy site at 2.5cm (%) | 94.73±3.87 | 94.83±3.80 | 0.903 |
| Coronal osteotomy site at 7.5cm (%) | 80.88±7.49 | 81.01±5.99 | 0.926 |
| Sagittal osteotomy site at 7.5cm (%) | 57.72±13.46 | 60.16±9.34 | 0.321 |
| Coronal osteotomy site at isthmus (%) | 74.45±14.17 | 74.06±13.23 | 0.893 |
| Sagittal osteotomy site at isthmus (%) | 51.34±14.05 | 52.89±13.16 | 0.590 |
| Femoral stem alignment (°) | 1.78±0.61 | 2.31±1.24 | 0.010* |
| Leg length discrepancy (mm) | 3.14±1.58 | 5.81±6.30 | 0.006** |
| **Posterior or lateral approach** | | | |
| HCOR (mm) | 0.46±1.18 | 1.49±7.97 | 0.535 |
| VCOR (mm) | -0.02±0.45 | 8.10±13.11 | 0.034* |
| Inclination (°) | 42.20±4.74 | 41.60±6.70 | 0.009* |
| Anteversion (°) | 18.34±2.63 | 18.13±3.31 | 0.810 |
| Coronal osteotomy site (%) | 61.48±7.81 | 59.82±6.95 | 0.439 |
| Sagittal osteotomy site (%) | 94.58±3.72 | 94.19±3.21 | 0.695 |
| Coronal osteotomy site at 2.5cm (%) | 74.65±10.20 | 74.26±8.39 | 0.886 |
| Sagittal osteotomy site at 2.5cm (%) | 93.89±3.35 | 93.02±4.07 | 0.423 |
| Coronal osteotomy site at 7.5cm (%) | 78.65±6.91 | 74.12±9.06 | 0.059 |
| Sagittal osteotomy site at 7.5cm (%) | 60.79±10.75 | 57.84±11.40 | 0.366 |
| Coronal osteotomy site at isthmus (%) | 73.55±8.78 | 67.09±18.05 | 0.123 |
| Sagittal osteotomy site at isthmus (%) | 58.68±10.83 | 52.72±16.02 | 0.140 |
| Femoral stem alignment (°) | 1.79±0.71 | 2.08±0.82 | 0.204 |
| Leg length discrepancy (mm) | 2.35±1.33 | 5.90±6.46 | 0.014* |

RAS-THA, robot-assisted total hip arthroplasty; M-THA, manual total hip arthroplasty; HCOR, horizontal displacement of the acetabular center of rotation; VCOR, vertical displacement of the acetabular center of rotation. * represents P<0.05, ** represents P<0.01.

eFigure1. Schematic diagram of measurement of radiographic parameters.


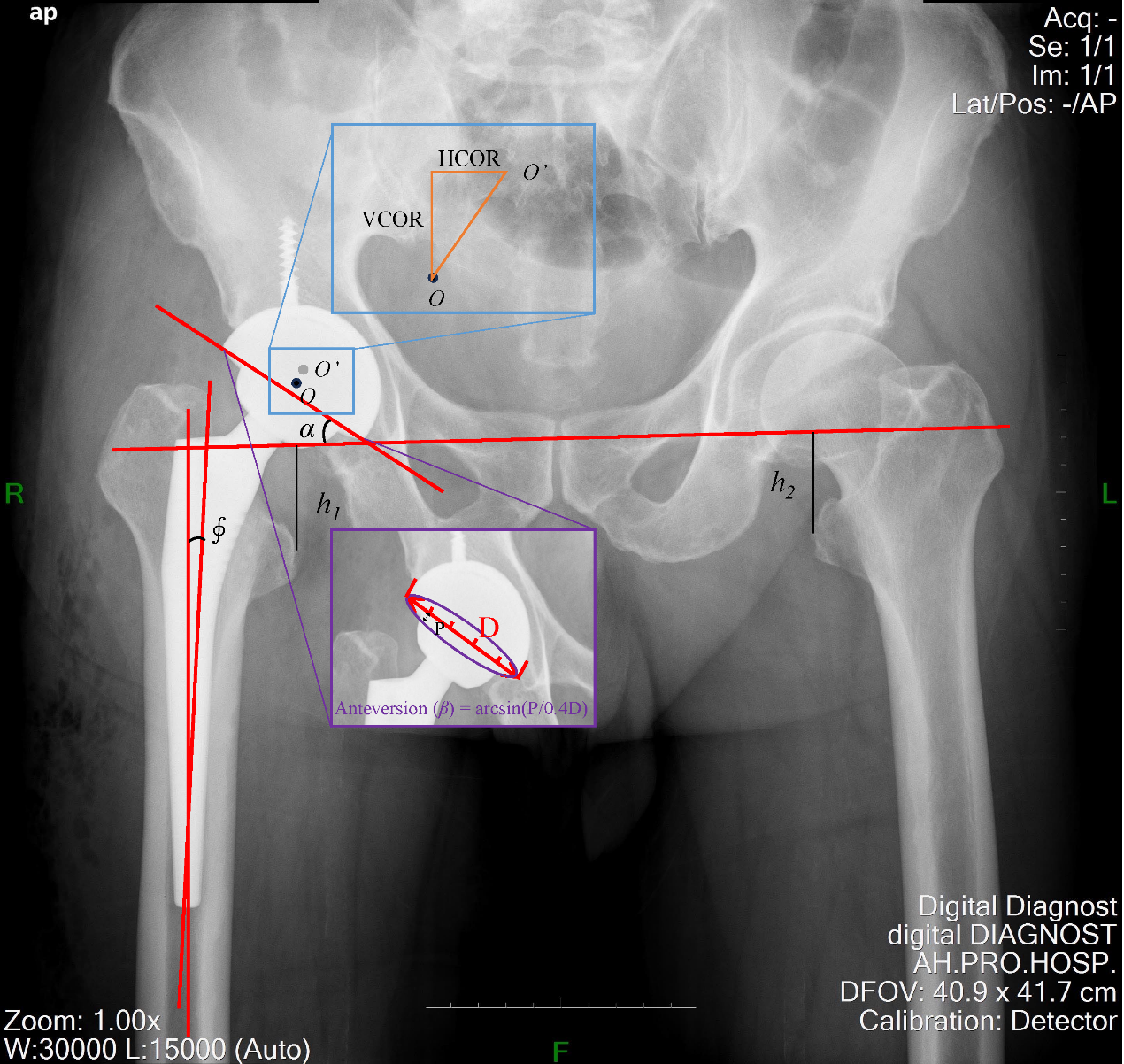


The angle between the line connecting the lowest points of the two ischial tuberosities and the long axis of the acetabular component was defined as acetabular component inclination (*α*). The metal edge of the acetabular component is projected as an ellipse on the AP radiograph. D is the length of the longer axis of the ellipse. Draw a perpendicular line intersecting the ellipse at one-fifth of the longer axis and measure its length P. The anteversion (*β*)is calculated as *β*= arcsin(P/0.4D) The angle between the long axis of the stem and the long axis of the femur was defined femoral stem alignment (*∮*). A line is made along the lowest point of the bilateral ischial tuberosity on the standard hip anteroposterior X-ray, and the vertical distance from the bilateral femoral trochanter to this line is considered as leg length discrepancy *(h_1_-h_2_*).
